# Immune thrombocytopenic purpura after influenza vaccine administration; a systematic review and meta-analysis

**DOI:** 10.1186/s40794-023-00206-9

**Published:** 2023-11-25

**Authors:** Mohamed Elsaid, Arvind Nune, Aml M. Brakat, Ayush Anand, Mahmoud Alashwah, Ahmed Maher, Nitu Lama, Criselle Angeline C. Peñamante

**Affiliations:** 1 Faculty of Medicine, Misr University for Science and Technology, 6th of October, Giza, Egypt; 2 Department of Rheumatology and General Medicine, Southport and Ormskirk Hospital NHS Trust, Southport, UK; 3 Faculty of Medicine, Zagazig University, Ash Sharqia Governorate, Egypt; 4 B. P. Koirala Institute of Health Sciences, Dharan, Nepal; 5 Faculty of Medicine, Cairo University, Cairo, Egypt; 6 Faculty of Medicine, Al-Azhar University, New-Damietta, Egypt; 7 Dr. M. V. Shetty College of Physiotherapy, Rajiv Gandhi University of Health Sciences, Mangaluru, India; 8 Department of Clinical Epidemiology, Faculty of Medicine and Surgery, University of Santo Tomas, Manila, Philippines; 9 Department of Psychology, College of Science, University of Santo Tomas, Manila, Philippines; 10 Medical Research Platform, Giza, Egypt

**Keywords:** Immune thrombocytopenia (ITP), Influenza vaccine, Platelets, Systematic review, Meta-analysis.

## Abstract

**Background:**

The American Society of Haematology defines immune thrombocytopenic purpura (ITP) as a common hematologic disorder characterized by a transient or long-term decrease in platelet counts (< 100 × 109/L.), purpura, and haemorrhagic episodes caused by antiplatelet autoantibodies, with the exclusion of other clinical conditions. We aimed to systematically determine the incidence of ITP in adults and children following influenza vaccination, the duration between vaccination and the occurrence of ITP, and to identify predictors of ITP after the vaccine.

**Methods:**

We searched PubMed, Cochrane Library, Google Scholar, Web of Science, Scopus, and Science Direct. We included primary studies that assessed the occurrence of immune thrombocytopenia in individuals who had received any influenza vaccine (primary or booster dose), regardless of the dosage, preparation, time of administration, or age of the participants. We excluded studies that were (a) Narrative, scoping, and umbrella reviews ;(b) studies with no accessible full text, abstract-only studies, or (c) Overlapping or unreliable data. The risk of bias in the included studies was assessed using the Joanna Briggs Institute (JBI) tool. We categorized studies for qualitative analysis based on study design. Descriptive statistics were used to summarize quantitative data, including the incidence of ITP after influenza vaccination.

**Results:**

Out of 729 articles retrieved from the database search, we included 24 studies. All patients identified and included in this systematic review presented with immune thrombocytopenia, determined by their platelet count. The period between vaccination and the occurrence of ITP ranged from (2:35 days). The mean duration was 13.5 days. The analysis revealed a statistically significant incidence rate ratio (IRR) = 1.85,95% CI [1.03–3.32] of ITP occurrence after 42 days.

**Conclusions:**

Influenza-associated ITP is uncommon, self-limiting, non-life-threatening, and curable. None of the patients reported having severe adverse events or death. Further studies are required to confirm the exact incidence of the ITP to better understand the pathophysiology of ITP development post-influenza vaccination.

**Supplementary Information:**

The online version contains supplementary material available at 10.1186/s40794-023-00206-9.

## Introduction

Immune thrombocytopenic purpura (ITP), also known as idiopathic thrombocytopenic purpura, is an acquired bleeding disorder in adults and children that is characterized by thrombocytopenia leading to bleeding episodes that range in severity from a purpuric rash to epistaxis and bleeding. In addition, patients may experience bleeding, such as intracranial and intestinal bleeding, in severe cases with a platelet count of < 20,000/uL [1]. ITP occurs with an incidence rate of 1.6 to 3.9 per 100,000 patient-years, which increases with age and has a slight female preponderance. The age-adjusted prevalence of ITP is estimated at 9.5 per 100,000 persons in the USA. In contrast, its annual incidence is estimated to be 2.68 per 100,000 in Northern Europe [2]. A bone marrow biopsy is used to confirm ITP and to rule out other common causes of thrombocytopenia [1].

Genetic variations may contribute to ITP susceptibility, although the cause is complex, poorly understood, and due to multifactorial causes. Autoantibody-induced platelet destruction reduces platelet synthesis, and T-cell abnormalities, such as Th1 polarization, Th17 overabundance, and regulatory T-cell deficiency, are possible reasons for immune dysregulation leading to immune thrombocytopenia [3].

Other factors associated with the pathogenesis of ITP are drugs, infections, cancers, autoimmune diseases, and vaccinations such as the influenza vaccine [4] [5]. Despite protective immunity, vaccines might induce autoimmune responses. Although the pathogenesis of influenza vaccine-induced thrombocytopenia is still unknown, the literature suggests that ITP risk increases following vaccination through the exact mechanism of anti-platelet autoantibodies formation by microbial infections. Vaccines stimulate protective immunity by mimicking body mechanisms; both live and inactivated vaccines cause the development of ITP [6] [7]. A systematic review (SR) described an association between the measles-mumps-rubella (MMR) vaccine and ITP in children. They concluded that only children with persistent or chronic ITP who must receive MMR require care [8]. In addition, surveillance systems revealed that vaccines, such as hepatitis A, diphtheria, tetanus, and pertussis (DTP), diphtheria, tetanus, acellular pertussis (DTaP), and varicella vaccines, were associated with ITP in Canada between 1992 and 2007 [9].

The severity of ITP, patients’ criteria, platelet count, bone marrow changes, and presence of antiplatelet antibodies vary between studies.

There is a scarcity of reliable data concerning the incidence of ITP following influenza vaccination. Surveillance system reports are prone to reporting bias because they depend on each country’s vaccination schedule. There is insufficient data on clinical outcomes such as bleeding manifestations and the development of chronic thrombocytopenia after the influenza vaccination. Therefore, we conducted a SR and meta-analysis of the available medical literature to calculate the incidence of ITP after influenza vaccination.

## Methods

### Study design

This SR and meta-analysis was conducted according to the PRISMA guidelines, as shown in (Supplementary Tables 1 and 2). The study protocol was previously registered with the International Prospective Register of Systematic Reviews (PROSPERO) database as CRD42022313947.

### Search strategy

We conducted a systematic literature search in six databases: PubMed, the Cochrane Library, Google Scholar, Web of Science, Scopus, and Science Direct in March 2022. The search terms were modified to match the databases. The data were independently extracted using a standardized Excel sheet containing the primary baseline characteristics and the outcomes of interest.

### Selection criteria

As per the study protocol, we included primary studies that reported the occurrence of ITP after influenza vaccination in adults or children. However, we excluded secondary studies, studies with no accessible full text, and abstracts. To ensure data quality, overlapping data were removed. We included all studies that met our criteria, regardless of age, country, language, or publication date.

### Study selection

Our search results were exported into EndNote (Thompson Reuter, USA) and then to CADIMA to detect and remove duplications. All included studies were imported into CADIMA for the title and abstract screening by two authors independently, and a senior reviewer was consulted to make the final decision in case of any inconsistency. Full-text screening of included studies was performed by two independent reviewers using our criteria, and any disagreements were resolved through a discussion.

### Data extraction

We developed a standardized extraction sheet based on the desired outcome to extract relevant data from the included studies; that assessed and answered our review questions. The extraction sheet was divided into two sections: one for case reports and case series and the other for other study designs. Two independent reviewers extracted all relevant data, and a consensus was reached on any differences after a discussion. If there was no agreement, a senior reviewer was consulted to make the final decision.

### Assessment of risk of bias

Three independent reviewers evaluated the quality of the included studies for bias using the Joanna Briggs Institute critical appraisal tool. The overall risk of bias was categorized into good, fair, or poor. However, there was no tool for assessing the risk of bias for self-controlled case series studies. Thus, they were not applicable for assessment.

### Analysis

Extracted data was cleaned and prepared for qualitative and quantitative analysis. The odds ratio was extracted and analysed to measure the burden of ITP after influenza vaccination compared to no exposure. The meta-analysis was performed using R software (version 4.1.3) and the (meta) package. A subgroup analysis was performed to assess different factors influencing the results. Heterogeneity was calculated and was considered significant when I^2^ was greater than 50% with a p-value < 0.05. A fixed effect model was used unless heterogeneity was significant. On the other hand, data that was unsuitable for statistical analysis was assessed qualitatively through table presentation and interpretation.

## Results

### Search results

**Fig. 1:**
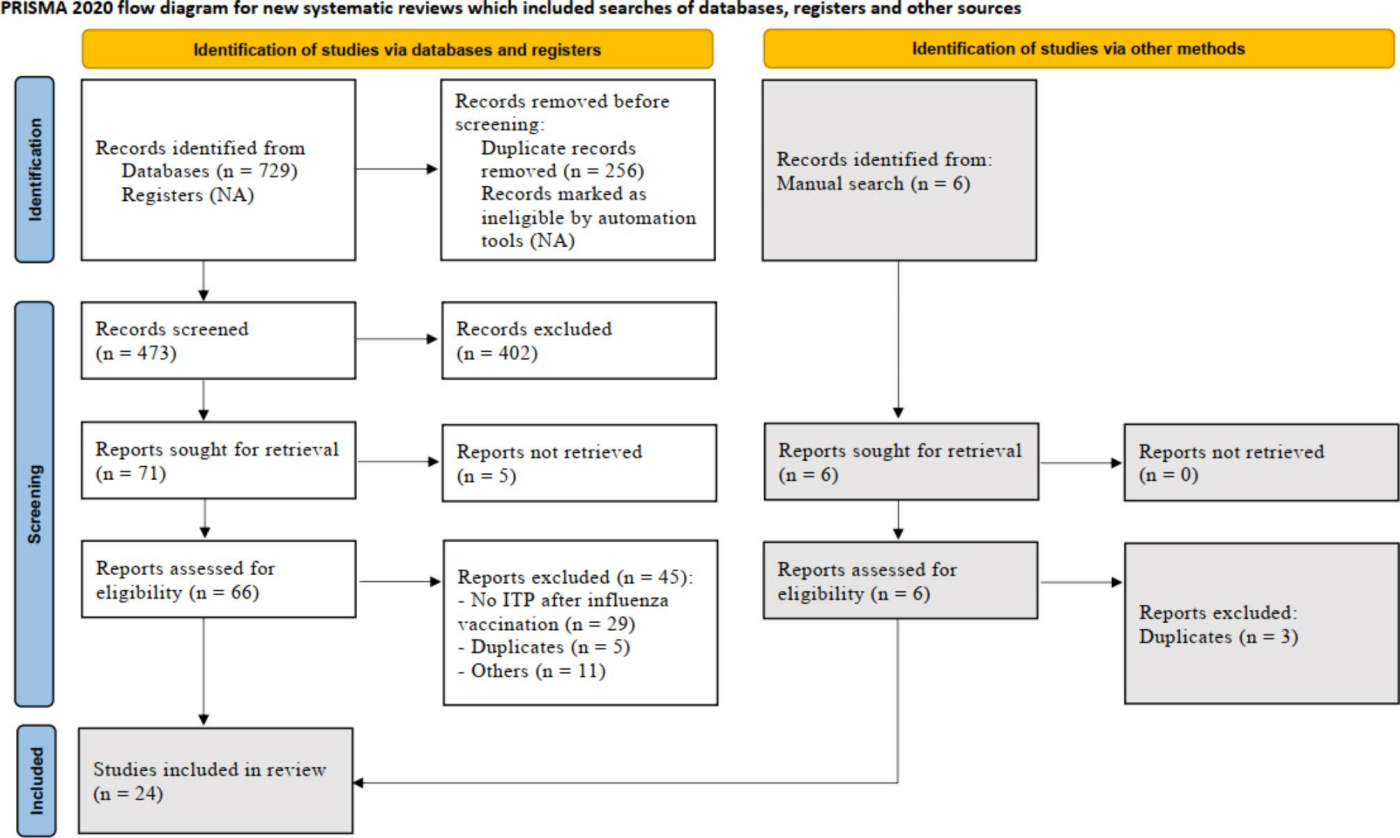
PRISMA flow diagram of included studies

The initial electronic database search yielded 729 studies; 71 met our inclusion criteria, and 21 were eligible after the full-text screening. A total of 45 studies were excluded by full-text screening, according to the PRISMA flow chart (Fig. 1**)**. A manual search yielded three additional studies. As a result, only 24 studies were included in our review. Additionally, a detailed illustration of the results of each database is provided in (Supplementary Table 3).

### Risk of bias assessment

Out of 24 included articles, 10 received good scores, 11 got fair and 3 self-controlled case series (SCCS) received no quality assessment. The score of each article is shown in Supplementary Tables (4, 5, 6, 7 and 8). We have included 1 RCT study with a good score. Out of 11 case report studies, 4 received a good score, and 7 received a fair score. Two Cohorts and 2 case controls received a good score. Of 5 cross sectional studies, 1 received a good score and other 4 got a fair score.

### Qualitative analysis of reported cases

#### Study characteristics

Eleven case series that comprised 13 patients were identified and described [10–20]. The mean age was 52.9 years (3–88), and 53.8% of patients were male. The most prevalent geographic location where thrombocytopenia was reported following influenza vaccination was Japan (6, 46%). Several patients presented with chronic diseases prior to receiving the influenza vaccine, including hypertension (2, 15%), primary biliary cirrhosis (1, 8%), and hepatitis C (1, 8%).

All patients had received an influenza vaccination; however, only 4 case series reported the name of the vaccine. Of these 4, 2 received Vaxigrip, and 1 received Fluarix, and 1 patient received Kitaken. Only 2 patients were co-administered with the influenza vaccination and other vaccines, including DTP/Polio/Hib, Hepatitis B, and pneumococcal conjugate vaccine. The ITP occurred following 1 dose of the influenza vaccine in 3 patients (23%), following three doses in 2 patients (15%), and following 2 doses in 1 patient (8%). The remaining 7 case reports did not present data on how many vaccine doses the patients received when ITP occurred (Table 1).

**Table 1:** Study characteristics of included case reports

Author (Year)	Age (Years)/ Gender (M/F)	Co-morbidity	Vaccination Type	Doses
Tishler et al. (2006)	68 M	Hypertension	Vaxigrip™	NR
Mamori et al. (2008)	75 F	Primary Biliary Cirrhosis	Influenza HA Vaccine ‘Kitaken’	NR
Kelton et al. (1981)	38 M	COPD, Bronchiectasis	NR	NR
Wan Jamaludin et al. (2018)	31 F	Hodgkin Lymphoma		1
Shlamovitz et al. (2013)	50 M	NR	NR	1
Ikegame et al. (2006)	19 F	Acute lymphoblastic leukaemia		1
Almohammadi et al. (2019)	68 M	Hepatitis C, prediabetes, hypertriglyceridemia	NR	3
Hamiel et al. (2016)	4.5 M	NR	Fluarix (manufactured by GlaxoSmithKline Biologicals, Dresden, Germany)	3
Mantadakis et al. (2010)	3 M	NR	(Vaxigrip, Sanofi Pasteur SA, Lyon, France)	2
Nagasaki et al. (2016)	81 F 75 F 87 F	NR	NR	NR
Ohta et al. (2022)	88 M	Stroke, Hypertension. Dyslipidaemia	NR	NR

#### ITP clinical presentation

All patients identified and included in this SR presented with ITP, diagnosed after examining their platelet count. Table 2 describes the platelet count before and after vaccination for each patient, with a substantial decrease being observed in all patients. The period between vaccination and the occurrence of ITP differed greatly between patients, ranging between 2 and 35 days. The mean duration was 13.5 days. Bone marrow biopsy findings were described in 8 cases, with hypercellularity observed in 5 cases (38%) (Table 2). Purpura or a bleeding event was described in 12 cases (92.3%), including purpuric rashes, epistaxis and petechiae, haemoptysis, bruising, buccal haematoma, bleeding gums, petechial lesions to the sclera, nasal bleeding, gross haematuria, cutaneous and mucosal bleeding, genital bleeding, and bleeding blisters.

**Table 2:** ITP clinical presentation in included cases

Author (Year)	Platelet Count		Bone marrow findings	Period between vaccination and occurrence of ITP (days)
	Before vaccine	After vaccine		
Tishler et al. (2006)	NR	3,000/mm^3^	hypercellularity with many megakaryocytes and no infiltration of foreign cells	14
Mamori et al. (2008)	164 × 10^3^/µL	5 × 10^3^/ml	NR	7
Kelton et al. (1981)	Reported to be normal	20,000 /µL	NR	14
Wan Jamaludin et al. (2018)	203 × 10^9^/L	3 × 10^9^/L	hypo-normocellularity, adequate megakaryocytopoiesis without lymphomatous relapse or dysplasia supportive of ITP	7
Shlamovitz et al. (2013)	NR	less than 5 k/mL	hypercellular marrow with erythroid and megakaryocytic hyperplasia. There was a mild to moderate left shift of the red cell precursors and megakaryocytes	4
Ikegame et al. (2006)	180 × 10^9^/l	3810 38 × 10^9^/l and 10 × 10^9^/l on days 14 and 17	Bone marrow examination revealed hyperplastic megakaryopoiesis, with no evidence of relapse	14
Almohammadi et al. (2019)	210,000–267,000/µL	0/µL	NR	2
Hamiel et al. (2016)	NR	17 000/µL	NR	7
Mantadakis et al. (2010)	NR	11,000/UL	NR	26
Nagasaki et al. (2016)	184,000/𝜇L	39,000/𝜇L	normocellular; the megakaryocyte count was 56/𝜇L	28
Ohta et al. (2022)	251,000/𝜇L	5,000/𝜇L	Bone marrow was normocellular.	35

### Quantitative analysis

A total of 13 studies [4, 13, 19, 21–30] reported the occurrence of ITP after influenza vaccination. Five [5, 27–29, 31]studies were surveillance representing 38.4% of included studies, while self-controlled case series were presented in 3 [13, 19, 25]studies. Cohort [24, 30]and case-control [4, 22] studies were conducted in 2 studies, while only 1 [23] study was a randomized controlled trial. More details regarding the country and type of influenza vaccine are provided in (Table 3).

**Table 3:** Study characteristics of included observational studies

Study	Study Design	Study Period	Country	Type of influenza vaccine
Huang et al. 2013	SCCS	2009–2010	Taiwan	monovalent MF59H adjuvanted vaccine /monovalent inactivated vaccine without adjuvant
Moro et al. 2013	Cohort	2009–2010	Italy	MF59-adjuvanted A/H1N1 influenza vaccine, Focetria®(Novartis Vaccines & Diagnostics, Siena, Italy)
O’Leary et al. 2011	SCCS	2000–2009	US	TIV
Woo et al. 2011	Cross sectional	1990–2008.	US	Live intranasal trivalent influenza virus
			US	Trivalent inactivated influenza virus vaccine (FLU)
Grimaldi-Bensouda et al. 2012	Case control	2008–2011	France	injectable/killed type
Isai et al. 2012	Cross sectional	2009–2010	European union	Adjuvanted (CelturaTM, Fluval PTM, FocetriaTM, PandemrixTM)
Lafaurie et al. 2022	SCCS	2009–2018	France	Influenza vaccine
Huand et al. 2012	Cross sectional	2009–2010	Taiwan	H1N1 Vaccine
Nakayama et al. 2007	Cross sectional	1994:2004	Japan	monovalent inactivated vaccine
Yokomichi et al. 2020	Case control	till 2019	Japan	NR
Clayes et al. 2018.	RCT	2011–2012	Belgium, Czech, Republic, Poland, Spain, United Kingdom	A/H1N1: California/7/2009§ A/H3N2: Victoria/210/2009 B Victoria: Brisbane/60/2008 B Yamagata: Brisbane/3/2007
		April 9, 2012 – Dec 12, 2012*	Bangladesh, Dominican Republic, Honduras	A/H1N1: California/7/2009§ A/H3N2: Victoria/210/2009 B Victoria: Brisbane/60/2008 B Yamagata: Hubei-Wujiagang/158/2009
		2012–2013	Belgium, Czech Republic, Lebanon, Poland, Spain, Turkey, UK	A/H1N1: Christchurch/16/2010§ A/H3N2: Victoria/361/2011 B Victoria: Brisbane/60/2008 B Yamagata: Hubei-Wujiagang/158/2009
		March 7, 2013 – Dec 2, 2013*	Bangladesh, Dominican Republic, Honduras, Philippines, Thailand	A/H1N1: Christchurch/16/2010§ A/H3N2: Victoria/361/2011 B Victoria: Brisbane/60/2008 B Yamagata: Hubei-Wujiagang/158/2009
		March 11, 2014 – Dec 31, 2014*	Bangladesh, Dominican Republic, Honduras, India, Philippines, Thailand	A/H1N1: Christchurch/16/2010§ A/H3N2: Texas/50/2012 B Victoria: Brisbane/60/2008 B Yamagata: Massachusetts/2/2012
Haber et al. 2015	Cross sectional	2013:2014	United States of America	LAIV4/LAIV3
Villa et al. 2013	Cohort	3 years	Italy	LAIV3

**Fig. 2:**
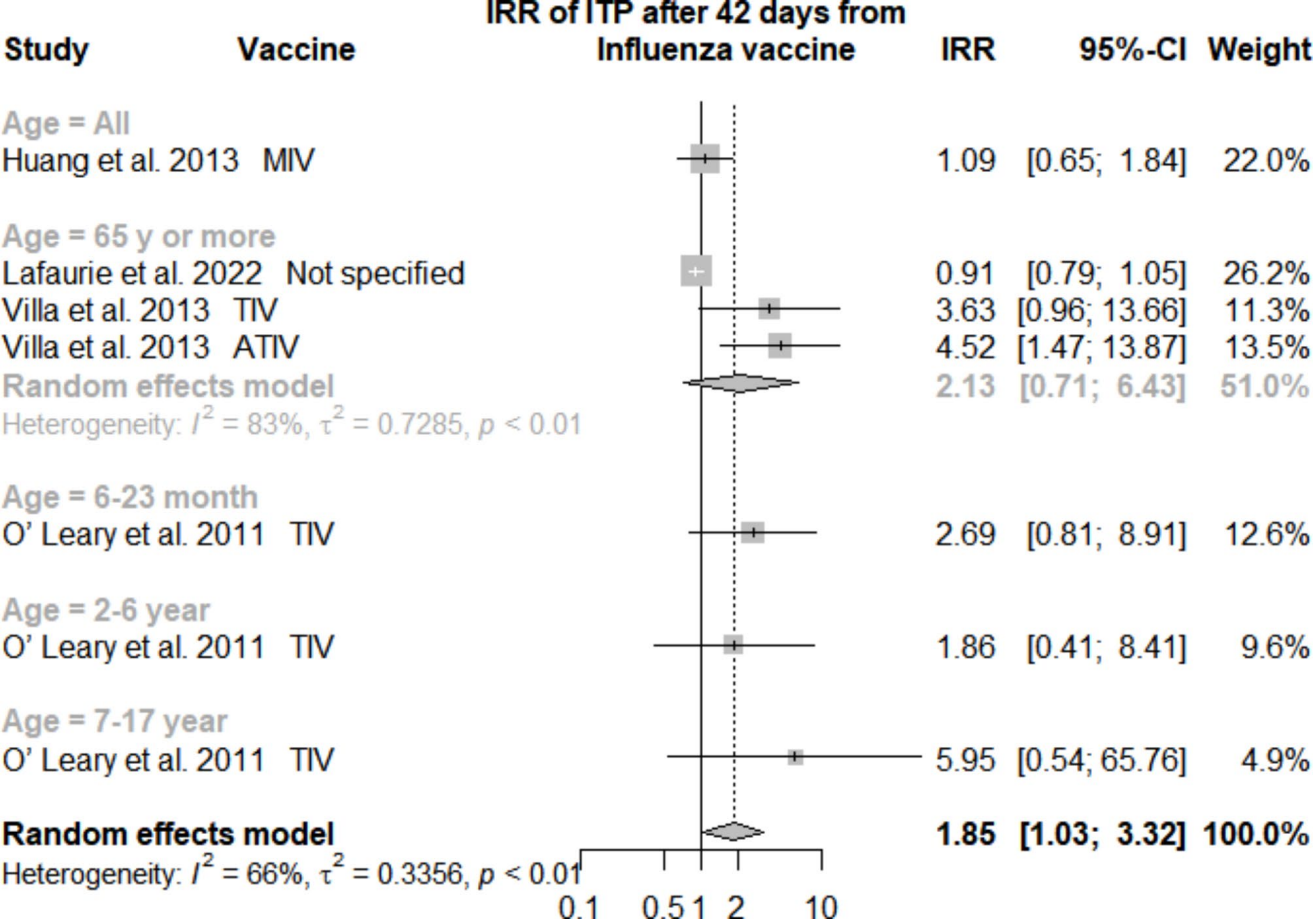
Meta-analysis of incidence rate ratio (IRR) of ITP after 42 days of influenza vaccine administration

Only 4 [24, 25, 27, 30] studies were included in the analysis of the incidence rate ratio (IRR) of ITP occurrence 42 days after receiving influenza vaccination. Generally, the analysis showed a positive statistically significant IRR = 1.85, 95% CI [1.03–3.32]. However, heterogeneity was statistically significant with I^2^ = 0.66% and a p-value < 0.01. Thus, a subgroup analysis according to the age of patients was performed, and it was only applicable in 3 studies [24, 25, 30]for the age group above 65 years. This yielded a statistically insignificant positive IRR = 2.13, 95% CI [0.71–6.43] with I^2^ = 83% and p-value < 0.01 (Fig. 2).

## Discussion

This study reviewed 24 articles on thrombocytopenia following the influenza vaccination. All 13 cases reported a drop in platelet count post-vaccination, indicative of ITP development. Furthermore, the majority of authors stated that the influenza vaccination was responsible for the onset of ITP, occurring at a mean duration of 13.5 days post-vaccination. A large proportion of cases were reported from Japan, and this geographical location was discussed among observational studies that investigated ITP development following other live vaccinations.

Few patients had chronic disease prior to vaccination, implying that thrombocytopenia developed irrespective of pre-existing conditions. ITP is characterised by its heterogeneous manifestations and unpredictable outcomes; however, the current literature suggests several risk factors for ITP development and chronic disease. ElAlfy et al. sought to identify the predictors for ITP in 409 patients with a confirmed ITP diagnosis. Several relevant risk factors were observed for the development of chronic ITP, specifically an initial platelet count of less than 20 × 109/L and a presentation age of more than ten years [32]. The risk of the development of ITP is increased depending on a patient’s platelet count, if they are female, and their exposure to NSAIDs [33].

The pathophysiology of vaccine-induced thrombocytopenia is poorly understood, with few large-scale research studies observing significant findings or concerning patterns. Haber et al. conducted an observational study investigating adverse outcomes following quadrivalent live attenuated influenza vaccination in the United States. The most common adverse events were neurologic conditions, including seizures and Guillain-Barré syndrome. Asthma and wheezing were also reported in a few children. However, no occurrence of ITP was reported among all 779 participants. Besides, no concerning patterns of adverse events were identified [34].

The cases identified in this SR reported on the occurrence of thrombocytopenia in adult patients. However, few studies reported on the incidence of ITP in this population. Moreover, this review focused exclusively on the development of ITP following the influenza vaccination, yet, current research extends to other live, inactivated, and simultaneous vaccinations. Yokomichi et al. assessed the risk of ITP following live, inactivated, and simultaneous vaccination in children under two years old. Despite the limitation, the findings suggest no significant ITP risk following single vaccinations or simultaneous vaccination spanning all age groups, including children [22].

Although detailed data was presented in case reports, a few observational studies are worth mentioning. Nakayama et al. investigated several cases of vaccine adverse events reported in the Kitasato Institute’s post-marketing surveillance, categorising patient responses into allergic reactions and severe systemic illnesses. The evidence concerning the relationship between thrombocytopenia and the influenza vaccination describes 12 cases of ITP. However, the occurrence of ITP was not significant, given the large sample size included in this report. This is reflected in the estimated incidence of severe neurological illness post-vaccination at 0.1–0.2 per million immunisation practices [5]. Additionally, Villa et al. discussed the safety of MF59-adjuvanted influenza vaccination in the elderly population of Northern Italy. Despite the fact that 170,998 vaccine doses were administered to 107,661 individuals during the study period, adverse events that requiring hospitalisation was rare. This emphasizes the safety of seasonal influenza vaccinations in older people [24].

Lafaurie et al. assessed the risk of ITP in a nationwide study in France, comprising 4394 patients with incident primary immune thrombocytopenia. The number of patients that had received at least one dose of the influenza vaccination was included. However, there was no increased risk of ITP following the influenza vaccine [30]. O’Leary et al., on the other hand, investigated the risk of ITP following childhood vaccines besides MMR amongst a cohort of 1.8 million children aged six weeks to 17 years. In total, 197 confirmed ITP cases were identified, indicating no elevated risk of ITP after any vaccine in early childhood. However, a significantly increased risk of ITP was observed following the hepatitis A vaccination in children aged seven to 17 years and for varicella and tetanus-diphtheria-acellular pertussis vaccination in adolescents aged 11 to 17 years [27].

Our SR has a few limitations. The review is based on published cases and observational studies. However, most cases of ITP are known to be asymptomatic [35] and, therefore, might not have made it to scientific reportage. It is possible that the cases we reviewed may not have been representative of the whole population. The influenza vaccine is usually administered on a priority basis which tends to skew towards the elderly and those with other comorbidities [36]. Therefore, the reviewed studies might have inadvertently left out a critical sample. Furthermore, because case reports were included, the level of evidence may be low. We may have missed some of the investigation results due to reporting bias. However, the bias was mitigated by strict inclusion and exclusion criteria and a comprehensive analysis of all included articles by multiple researchers. Due to the small number of reported cases, it was impossible to compare the characteristics and severity of ITP across regions. Meta-analysis was not conclusive because of heterogeneity.

## Conclusion and future directions

This SR corroborated that ITP post-influenza vaccinations is rare. Several observational studies also substantiate the safety profile of influenza vaccinations. None of the patients reported having severe adverse events or death. More research is required to ascertain the true incidence and pathophysiology of ITP post-influenza vaccination. Further large-scale prospective studies are required to establish the characteristics and risk factors of influenza vaccine‐associated ITP. Further research is also needed to explore the influenza vaccine’s causal role in the development of ITP by investigating epitope similarities between platelets and vaccine‐driven antigens. The potential disparity between the different influenza vaccine brands to induce ITP warrants further exploration and may aid in optimizing vaccine production to produce vaccines with a lower risk of triggering autoimmunity.

### Electronic supplementary material

Below is the link to the electronic supplementary material.

Supplementary Material 1

## Data Availability

Data is provided in the manuscript while codes are available upon reasonable request.

## References

[1] Zufferey A, Kapur R, Semple J. Pathogenesis and Therapeutic Mechanisms in Immune Thrombocytopenia (ITP). J Clin Med [Internet]. 2017;6(2):16. Available from: http://www.mdpi.com/2077-0383/6/2/16.

[2] Michel M (2009). Immune thrombocytopenic purpura: epidemiology and implications for patients. Eur J Haematol.

[3] Georgi JA, Middeke JM, Bornhäuser M, Matzdorff A, Trautmann-Grill K (2023). Deciphering the genetic basis of immune thrombocytopenia: current evidence for genetic predisposition in adult ITP. Blood Adv.

[4] Grimaldi-Bensouda L, Michel M, Aubrun E, Leighton P, Viallard JF, Adoue D (2012). A case-control study to assess the risk of immune thrombocytopenia associated with vaccines. Blood.

[5] Nakayama T, Onoda K (2007). Vaccine adverse events reported in post-marketing study of the Kitasato Institute from 1994 to 2004. Vaccine.

[6] Schattner A, Schattner A. 2005. “Consequence or Coincidence?” Vaccine 23(30): 3876–86. https://linkinghub.elsevier.com/retrieve/pii/S0264410X05003506.Consequence or coincidence? Vaccine. 2005;23(30):3876–86. 10.1016/j.vaccine.2005.03.005 15917108

[7] Perricone C, Ceccarelli F, Nesher G, Borella E, Odeh Q, Conti F (2014). Immune thrombocytopenic purpura (ITP) associated with vaccinations: a review of reported cases. Immunol Res.

[8] Cecinati V, Principi N, Brescia L, Giordano P, Esposito S (2013). Vaccine administration and the development of immune thrombocytopenic purpura in children. Hum Vaccin Immunother.

[9] Sauvé LJ, Scheifele D (2009). Do childhood vaccines cause thrombocytopenia?. Paediatr Child Health.

[10] Wan Jamaludin WF, Kok WH, Loong L, Palaniappan SK, Zakaria MZ, Ong TC (2018). Vaccine-induced immune thrombocytopaenia purpura in autologous haematopoietic stem cell transplantation. Med J Malaysia.

[11] Shlamovitz GZ, Johar S (2013). A case of Evans’ syndrome following influenza vaccine. J Emerg Med.

[12] Ikegame K, Kaida K, Fujioka T, Kawakami M, Hasei H, Inoue T (2006). Idiopathic thrombocytopenic purpura after influenza vaccination in a bone marrow transplantation recipient [7]. Bone Marrow Transplant.

[13] Kelton JG (1981). Vaccination-Associated Relapse of Immune Thrombocytopenia. JAMA: The Journal of the American Medical Association.

[14] Almohammadi A, Lundin MS, Abro C, Hrinczenko B (2019). Epistaxis and gross haematuria with severe thrombocytopaenia associated with influenza vaccination. BMJ Case Rep.

[15] Hamiel U, Kventsel I, Youngster I (2016). Recurrent immune thrombocytopenia after influenza vaccination: a case report. Pediatrics.

[16] Mantadakis E, Farmaki E, Thomaidis S, Tsalkidis A, Chatzimichael A (2010). A case of Immune Thrombocytopenic Purpura after Influenza Vaccination. J Pediatr Hematol Oncol.

[17] Mamori S, Amano K, Kijima H, Takagi I, Tajiri H (2008). Thrombocytopenic purpura after the administration of an influenza vaccine in a patient with autoimmune liver disease. Digestion.

[18] Tishler M, Levy O, Amit-Vazina M (2006). Immune thrombocytopenic purpura following influenza vaccination. Isr Med Association J.

[19] Nagasaki J, Manabe M, Ido K, Ichihara H, Aoyama Y, Ohta T (2016). Postinfluenza Vaccination Idiopathic Thrombocytopenic Purpura in Three Elderly Patients. Case Rep Hematol.

[20] Ohta R, Sano C (2022). Severe Immune Thrombocytopenic Purpura following influenza vaccination: a Case Report. Cureus.

[21] 102-. Vaccine adverse events reported in post-marketing study of the Kitasato Institute from 1994 to 2004_Nakayama _2007.pdf. 10.1016/j.vaccine.2006.05.130 16945455

[22] Yokomichi H, Tanaka-Taya K, Koshida R, Nakano T, Yasui Y, Mori M (2020). Immune thrombocytopenic purpura risk by live, inactivated and simultaneous vaccinations among japanese adults, children and infants: a matched case–control study. Int J Hematol.

[23] Claeys C, Zaman K, Dbaibo G, Li P, Izu A, Kosalaraksa P (2018). Prevention of vaccine-matched and mismatched influenza in children aged 6–35 months: a multinational randomised trial across five influenza seasons. Lancet Child Adolesc Health.

[24] Villa M, Black S, Groth N, Rothman KJ, Apolone G, Weiss NS (2013). Safety of MF59-adjuvanted influenza vaccination in the elderly: results of a comparative study of mf59-adjuvanted vaccine versus nonadjuvanted influenza vaccine in Northern Italy. Am J Epidemiol.

[25] Huang WT, Yang HW, Liao TL, Wu WJ, Yang SE, Chih YC et al. Safety of pandemic (H1N1) 2009 Monovalent Vaccines in Taiwan: a self-controlled Case Series Study. PLoS ONE. 2013;8(3). 10.1371/journal.pone.0058827 PMC3594153 23536827

[26] Moro ML, Nobilio L, Voci C, Di Mario S, Candela S, Magrini N (2013). A population based cohort study to assess the safety of pandemic influenza vaccine Focetria® in Emilia-Romagna region, Italy-Part two. Vaccine.

[27] O’Leary ST, Glanz JM, McClure DL, Akhtar A, Daley MF, Nakasato C (2012). The risk of immune thrombocytopenic purpura after vaccination in children and adolescents. Pediatrics.

[28] Woo EJ, Wise RP, Menschik D, Shadomy SV, Iskander J, Beeler J (2011). Thrombocytopenia after vaccination: Case reports to the US vaccine adverse event reporting system, 1990–2008. Vaccine.

[29] Isai A, Durand J, Le Meur S, Hidalgo-Simon A, Kurz X (2012). Autoimmune disorders after immunisation with Influenza A/H1N1 vaccines with and without adjuvant: EudraVigilance data and literature review. Vaccine.

[30] Lafaurie M, Lapeyre-Mestre M, Sailler L, Sommet A, Moulis G. Risk of Immune Thrombocytopenia After Influenza Vaccine. JAMA Intern Med [Internet]. 2022; Available from: https://jamanetwork.com/journals/jamainternalmedicine/fullarticle/2788998. 10.1001/jamainternmed.2021.8523 PMC8861894 35188544

[31] Haber P, Moro PL, Cano M, Lewis P, Stewart B, Shimabukuro TT (2015). Post-licensure surveillance of quadrivalent live attenuated influenza vaccine United States, Vaccine adverse event reporting system (VAERS), July 2013-June 2014. Vaccine.

[32] ElAlfy M, Farid S, Maksoud AA (2010). Predictors of chronic idiopathic thrombocytopenic purpura. Pediatr Blood Cancer.

[33] Piel-Julian ML, Mahévas M, Germain J, Languille L, Comont T, Lapeyre-Mestre M (2018). Risk factors for bleeding, including platelet count threshold, in newly diagnosed immune thrombocytopenia adults. J Thromb Haemost.

[34] Haber P, Moro PL, Lewis P, Woo EJ, Jankosky C, Cano M (2016). Post-licensure surveillance of quadrivalent inactivated influenza (IIV4) vaccine in the United States, Vaccine adverse event reporting system (VAERS), July 1, 2013-May 31, 2015. Vaccine.

[35] Zitek T, Weber L, Pinzon D, Warren N (2022). Assessment and Management of Immune Thrombocytopenia (ITP) in the Emergency Department: current perspectives. Open Access Emergency Medicine.

[36] Straetemans M, Buchholz U, Reiter S, Haas W, Krause G (2007). Prioritization strategies for pandemic influenza vaccine in 27 countries of the European Union and the Global Health Security Action Group: a review. BMC Public Health.

